# Increased cortical expression of the zinc transporter SLC39A12 suggests a breakdown in zinc cellular homeostasis as part of the pathophysiology of schizophrenia

**DOI:** 10.1038/npjschz.2016.2

**Published:** 2016-03-09

**Authors:** Elizabeth Scarr, Madhara Udawela, Mark A Greenough, Jaclyn Neo, Myoung Suk Seo, Tammie T Money, Aradhana Upadhyay, Ashley I Bush, Ian P Everall, Elizabeth A Thomas, Brian Dean

**Affiliations:** 1 The Molecular Psychiatry Laboratory, The Florey Institute of Neuroscience and Mental Health, Parkville, VIC, Australia; 2 Department of Psychaitry, University of Melbourne, Melbourne, VIC, Australia; 3 CRC for Mental Health, Carlton South, VIC, Australia; 4 Oxidation Biology Laboratory, The Florey Institute of Neuroscience and Mental Health, Parkville, VIC, Australia; 5 Department of Medicine, Royal Melbourne Hospital, University of Melbourne, Melbourne, VIC, Australia; 6 Department of Molecular and Cellular Neuroscience, The Scripps Research Institute, La Jolla, CA, USA

## Abstract

Our expression microarray studies showed messenger RNA (mRNA) for solute carrier family 39 (zinc transporter), member 12 (*SLC39A12*) was higher in dorsolateral prefrontal cortex from subjects with schizophrenia (Sz) in comparison with controls. To better understand the significance of these data we ascertained whether *SLC39A12* mRNA was altered in a number of cortical regions (Brodmann’s area (BA) 8, 9, 44) from subjects with Sz, in BA 9 from subjects with mood disorders and in rats treated with antipsychotic drugs. In addition, we determined whether inducing the expression of *SLC39A12* resulted in an increased cellular zinc uptake. *SLC39A12* variant 1 and 2 mRNA was measured using quantitative PCR. Zinc uptake was measured in CHO cells transfected with human *SLC39A12* variant 1 and 2. In Sz, compared with controls, *SLC39A12* variant 1 and 2 mRNA was higher in all cortical regions studied. The were no differences in levels of mRNA for either variant of *SLC39A12* in BA 9 from subjects with mood disorders and levels of mRNA for *Slc39a1*2 was not different in the cortex of rats treated with antipsychotic drugs. Finally, expressing both variants in CHO-K1 cells was associated with an increase in radioactive zinc uptake. As increased levels of murine *Slc39a12* mRNA has been shown to correlate with increasing cellular zinc uptake, our data would be consistent with the possibility of a dysregulated zinc homeostasis in the cortex of subjects with schizophrenia due to altered expression of *SLC39A12*.

## Introduction

Schizophrenia is increasingly acknowledged as occurring in individuals with a genetic predisposition and the process toward frank illness is triggered by an encounter with as yet unknown environmental factors.^1^ It is now known that environmental factors act through a variety of epigenetic mechanisms to modulate gene expression^2^ and it is proposed that the interaction between genes and environment occur through epigenetic mechanisms, which act to change levels of gene expression. Therefore, it would be expected that alterations of gene expression makes a significant contribution to the underlying pathophysiology of the disorders that result from gene×environment interactions.^3,4^ The hypothesis that altered gene×environment interactions contribute to the onset of schizophrenia by changing gene expression in the central nervous system (CNS) is supported by the extensive changes, measured by gene expression arrays, in postmortem CNS from subjects with the disorder.^5^ These data have given new insights into how changes in gene expression can affect biological pathways in the CNS to bring about the functional changes that precipitate the symptoms associated with schizophrenia. However, to increase our understanding of this new knowledge it is necessary to conduct more focused studies of individual genes, or a limited number of genes within defined biochemical pathways. These studies are needed to understand the full extent of changes in expression of a gene or genes in the CNS from subjects with schizophrenia and to gain an understanding of the biological processes modulated by the expression of that gene in specific CNS regions. By combining these data it will be possible to predict the functional consequences of changed gene expression in the human CNS.^5^

We have completed expression microarray studies in Brodmann’s area (BA) 9 (ref. 6) and 46.^7^ In the study in BA 9 from subjects with schizophrenia the most differential expressed gene (increased) was *SLC39A12* and, encouragingly, levels of messenger RNA (mRNA) for that gene was also significantly increased in BA 46 from subjects with the disorder. These data suggested that there were widespread changes in cortical *SLC39A12* expression in subjects with schizophrenia that could be contributing to changes in the many cortical functions, which are known to be altered in subjects with schizophrenia.^8^ Thus, in line with the notion that a better understanding of the data from studies in the human transcriptome requires more focused studies,^5^ we began to determine the extent of changes in *SLC39A12* expression in schizophrenia whether these changes in gene expression showed any diagnostic specificity or were part of the mechanisms of action of drugs used to treat the disorder.

On the basis of sequence homology, the human *SLC39A12* gene has been included as a member of a family of zinc (Zn) transporters designated as the ZRT-IRT-like proteins (current designation *SLC39*).^9^ ZRT-IRT-like proteins act in concert with another family of transporters, known in non-mammalian organisms as cation diffusion facilitators and in mammals as zinc transporters (ZnT),^10^ to regulate the distribution of Zn in the extra- versus intracellular milieu and within cellular compartments.^11^ Hence the ZnT maintain Zn homeostasis that is critical for the normal functioning of tissues including the CNS.^12^ Relevant to our studies, *SLC39A12* is highly expressed in the brain relative to peripheral tissues,^13^ which suggests it could be important in maintaining zinc homeostasis in the CNS.

Influencing our approach to studying *SLC39A12* in the cortex of subjects with schizophrenia was our discovery that a sub-set of subjects (25%) with the disorder have a marked decrease in the cortex muscarinic M1 receptor (CHRM1)^14^ that allows them to be separated into a discrete group that we have termed Muscarinic Receptor Deficit Schizophrenia (MRDS). We identified MRDS because of a marked loss of radioligand binding to the cortical CHRM1 and this is pertinent because we have now shown that Zn potently regulates the orthosteric binding site on CHRM1.^15^ This means the changes we see in CHRM1 could, at least in part, be owing to changes in the action of Zn on that receptor in MRDS. Other data supporting the interaction between CHRMs and Zn are those showing Zn administration increases the density of CHRM in rat CNS^16^ and, conversely, that CHRM1 has a role in controlling Zn uptake in differentiated neuroblastoma cells.^17^ Importantly, the interaction between Zn and CHRMs forms part of a much wider roles for Zn that include modulating such important CNS functions as glutamatergic neurotransmission through NMDA receptors, long term potentiation and synaptic plasticity.^12^ It is likely that changes in *SLC39A12* expression in the cortex of subjects with schizophrenia would affect functionality through many of these processes as well as influencing the functioning of CHRM1.

Given the potential for changes in *SLC39A12* expression to affect CNS function we decided to begin extending our expression array data by determining whether the expression of the two known variants of *SLC39A12,* variant 1 (NP_001138667.1) and variant 2 (NP_689938.2), were altered in BA 8 (frontal eye field), BA 9 (DLPFC) and BA 44 (part of Broca’s area) from subjects with schizophrenia. To take this understanding beyond pathophysiology at the level of the syndrome, our cohort of subjects with schizophrenia were made up of MRDS and non-MRDS. To gain the data on whether changes in *SLC39A12* expression might be specific to schizophrenia we measured levels of mRNA for the gene in BA 9 from subjects with major depressive disorder (MDD) and bipolar disorder (BD). To determine whether changes in *SLC39A12* expression could be an outcome of the mechanisms of action of antipsychotic drugs we measured levels of *SLC39A12* mRNA in the cortex of rats treated for 12 months with either antipsychotic drugs or vehicle. Finally, to begin to understand the function of variants of *SLC39A12*, beyond that suggested by gene sequence homology, we determine whether expression of the gene was associated with the increase in Zn uptake that would be expected with increased expression of a Zn transporter.

## Results

### Demographic, CNS collection, and dug histories

Compared to controls, brain pH was slightly, (−1.6%), but significantly lower in schizophrenia (*P*=0.030); there were no other significant differences in demographic, CNS collection or drug history data (Table 1). When subjects with schizophrenia were divided into MRDS and non-MRDS there were no significant variations in any demographic, tissue collection, or pharmacological related data.

In comparison with the controls and bipolar disorders, brain pH was higher (4.7%) in major depressive disorder (Table 1); there were no significant variations in any demographic, other tissue collection or pharmacological related data.

### *SLC39A12* variant 1 mRNA

**Figure 1:**
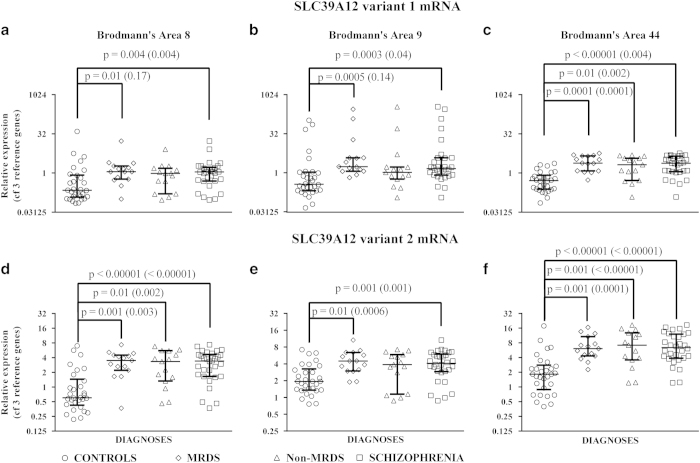
Relative levels of *SLC39A12* variant 1 (**a**, **b** and **c**) and variant 2 (**d**, **e** and **f**) mRNA (median±interquartile range) in BA 8 (**a** and **d**), 9 (**b** and **e**) and 44 (**c** and **f**) from subjects with schizophrenia and age-/sex-matched control subjects as well as subjects with schizophrenia divided into MRDS and non-MRDS. Probability values in brackets were obtained after analyzing *SLC39A12* variant 1 and 2 with potential confounds included as covariates MDRS, muscarinic receptor deficit schizophrenia; mRNA, messenger RNA.

Compared with controls, levels of *SLC39A12* variant 1 mRNA were higher in all three cortical regions from subjects with schizophrenia (BA 8: *P*=0.004, Figure 1a; BA 9: *P*=0003, Figure 1b and BA 44: *P*<0.00001; Figure 1c).

When the subjects with schizophrenia were divided into MRDS, non-MRDS and compared with controls, in BA 8 there was significant variation in *SLC39A12* variant 1 mRNA between groups (KW=9.2, *P*=0.01) with *post hoc* tests showing this was due to higher levels of *SLC39A12* variant 1 mRNA in the MRDS group compared with controls (*P*=0.01; Figure 1a). In BA 9, there was significant variation in *SLC39A12* variant 1 mRNA between groups (KW=15.4, *P*=0.00001) due to higher levels of *SLC39A12* variant 1 mRNA in the MRDS, but not non-MRDS, compared with controls (*P*=0.0005; Figure 1b). In BA 44, there was a significant variation in *SLC39A12* variant 1 mRNA (KW=23.9, *P*<0.0001) due to higher levels of *SLC39A12* variant 1 mRNA in both MRDS (*P*=0.0001) and non-MRDS (*P*=0.01; Figure 1c).

Compared with controls, levels of *SLC39A12* variant 1 mRNA did not vary with diagnosis in BA 9 from subjects with mood disorders (KW=3.1, *P*=0.21, Table 2).

### *SLC39A12* variant 2 mRNA

Compared with controls, levels of *SLC39A12* variant 2 mRNA were higher in all three cortical regions from subjects with schizophrenia (BA 8: *P*<0.00001, Figure 1d; BA 9: *P*=0.001, Figure 1e; and BA 44: *P*<0.00001, Figure 1f).

When the subjects with schizophrenia were divided into MRDS, non-MRDS, and compared with controls, in BA 8 there was a significant variation in *SLC39A12* variant 2 mRNA levels (KW=17.2, *P*=0.0002) due to higher levels of *SLC39A12* variant 2 mRNA in both schizophrenia MRDS (*P*=0.001) and non-MRDS (*P*=0.01) compared with controls (Figure 1d). In BA 9, there was variation in levels of *SLC39A12* variant 2 mRNA between groups (KW=11.0, *P*=0.004) due to higher levels of *SLC39A12* variant 2 mRNA in the MRDS subjects (*P*=0.01) compared with controls (Figure 1e). In BA 44, there were significant variations in *SLC39A12* variant 2 mRNA levels (KW=22.3, *P*<0.0001) due to higher levels of *SLC39A12* variant 2 mRNA in MRDS (*P*=0.001) and non-MRDS (*P*=0.001; Figure 1f).

Compared with controls, levels of *SLC39A12* variant 2 mRNA in BA 9 did not vary with diagnosis in mood disorders (KW=0.7, *P*=0.69; Table 2).

### Preclinical neuropsychopharmacology

Compared with vehicle, there was no significant variations in levels of rat cortical *Slc39a12* mRNA levels following treatment with haloperidol, chlorpromazine, or thioridazine (KW=1.99; *P*=0.57; Table 3).

### Potential confounds

Correlations between levels of *SLC39A12* variant 1 and 2 mRNA and CNS pH in each cortical region, both variants of *SLC39A12* mRNA with postmortem interval in BA 44 as well as *SLC39A12* variant 1 mRNA with RNA integratory number (RIN) and *SLC39A12* variant 2 mRNA with LEAP in BA 9 suggested these non-experimental variables as potential confounds (Supplementary Table S1). However, when levels of *SLC39A12* mRNA data were reanalysed with CNS pH, postmortem interval, RIN, and LEAP being included as covariates as appropriate, all significant differences with diagnoses remained except for *SLC39A12* variant 1 mRNA in BA 8 and 9 for MRDS compared with controls (Figure 1a and b).

In mood disorders, there was a correlation between CNS pH and levels of *SLC39A12* variants 1 and 2 mRNA (Supplementary Table S2). When levels of *SLC39A12* variant 1 and 2 mRNA were compared across diagnoses with CNS pH as a covariate levels of mRNA still did not vary with diagnoses (Variant 1 *P*=0.15; Variant 2 *P*=0.69).

### Zinc uptake studies

**Figure 2:**
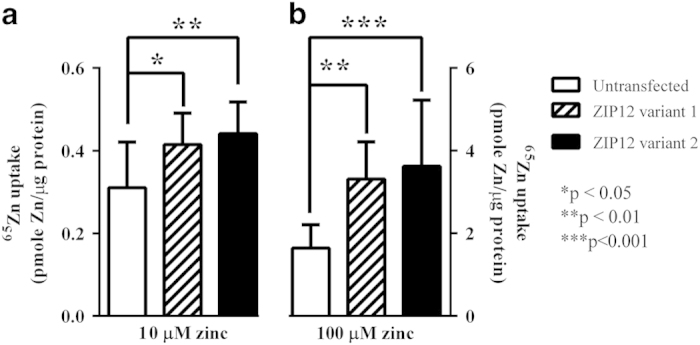
^65^Zinc accumulation in non-transfected CHO-K1 cells and those expressing recombinant human *SLC39A12* variant 1 (**a**) or 2 (**b**) following incubation with 10 and 100 μmol/l zinc for 10 min.

When CHO-K1 cells were transfected with *SLC39A12* variant 1 and variant 2 both variants were expressed with equal efficiency. There were dose-dependent increases in ^65^Zn uptake when the radioactive Zn was present at 10 μmol/l (F_2,30_=6.67, *P*=0.004) and 100 μmol/l (F_2,33_=11.04, *P*=0.0002). Under both conditions, cells that expressed either variant had higher radioactive Zn accumulation than controls (33% increase, *P*<0.05; Figure 2) and there was no difference in radioactive Zn accumulation between variant 1 and 2 expressing CHO-K1 cells.

## Discussion

At the functional level, this study is the first to show that increasing the level of expression of human *SLC39A12* variant 1 and 2 in CHO cells is associated with a significant increase in Zn transport. These data give experimental support to the proposal, based on gene sequence homology, that the *SLC39A12* gene encodes a Zn transporter. Our data complement those from a mouse study that reports the levels of murine *Slc39a12* mRNA in a cell that correlates with the level of Zn uptake.^13^

Our data from the human cortex adds to our expression array studies in showing that *SLC39A12* mRNA levels are higher in schizophrenia. This study extends our expression array findings by showing that mRNA for *SLC39A12* variant 1 and 2 are higher in BA 8, 9 and 44 from subjects with schizophrenia. However, our data on levels of *SLC39A12* variant 1 mRNA in BA 8 and 9 from subjects with MRDS need to be interpreted with caution as differences in levels of this mRNA may be due, in part, to the effects of CNS pH (BA 8 and 9) and RNA quality (RIN: BA 9). However, overall our data are consistent with the conclusion that there are widespread increases in the expression of *SLC39A12* in the cortex from subjects with schizophrenia some of which are limited to the cortex from subjects with MRDS.

Our study also shows that changes in *SLC39A12* variant 1 and 2 expression have some degree of diagnostic specificity as there was no change in levels of expression of either variant of *SLC39A12* in BA 9 from subjects with mood disorders.

Our current data also argue that changes in cortical levels of *SLC39A12* in schizophrenia are not simply due to antipsychotic drug treatment. This is because, levels of neither variant of *SLC39A12* mRNA varied with (i) the type or dose of antipsychotic drug prescribed to subjects with schizophrenia prior to death, (ii) in the cortex of rats after treatment with antipsychotic drugs used to treat the subjects with schizophrenia from whom tissue was obtained for this study or (iii) in expression array studies using cortex from rodents after typical and atypical antipsychotic drug treatment (rat^18,19^ and mouse;^20,21^ note expression arrays did have probes for *Slc39a12*). In addition, levels of *SLC39A12* mRNA only varied in subjects with MRDS who had similar antipsychotic drug treatments, whereas subjects with non-MRDS who argue against the changes in *SLC39A12* mRNA being simply an effect of drug treatment. Importantly, at the time of designing our studies it was proposed that rodents only expressed a single *Slc29a12* gene product. It has subsequently been shown that like humans, at least cows, rats, and mice express two forms of *Slc29a12.*^22^ The quantitative PCR (qPCR) primers we used in our study would amplify both forms of *Slc29a12* mRNA present in the rat cortex and therefore further experiments would be needed to determine whether antipsychotic drug treatment selectively affects the levels of mRNA for one of the two form of *Slc29a12* in rat cortex.

The hypothesis that changes in *SLC39A12* in the cortex of subjects with schizophrenia would be associated with changes in Zn transport is supported by the data that suggest the members of the ZRT-IRT-like proteins family are located in the plasma membrane, and increase intracellular Zn levels by facilitating uptake from the extracellular environment.^23^ There would appear to be a dynamic relationship between the localization of Zn and the activity of SLC39A12, because it has been reported that depleting intracellular Zn increases expression of *Slc39a12* mRNA in cultured murine T cells^23^ and the recruitment of Slc39a12 to the plasma membrane.^13^ Notably, SLC39A12 is the most highly expressed Zn transporter in the brain where it appears to have a pivotal role in the development of the neural network.^13^ At present, it is not clear as to what the consequences of increased expression of *SLC39A12* would be in the adult cortex.

Importantly, there is no evidence that changes in *SLC39A12* expression would impact on total cortical Zn levels, which means our findings on *SLC39A12* expression and the finding that total levels of Zn are not altered in the brains from subjects with schizophrenia^24–27^ are not conflicting. Current understanding of the roles of the multiple human ZnT is that not only are they critical in ensuing a homeostasis between extra- and intracellular Zn, but they also have a role in ensuing correct Zn homeostasis across cellular compartments.^11^ This could mean that changes in *SLC39A12* expression would most likely result in a redistribution of Zn within cellular compartments. Unfortunately, as yet, we do not have the capacity to test this hypothesis by measuring Zn in cellular compartments in human postmortem CNS tissue. However, animal studies have shown that changes in Zn homeostasis brought about by acute Zn depletion^28,29^ is associated with impaired spatial learning, whereas developmental Zn deficiency^30,31^ results in working memory deficits. Although these are deficits that are often seen in subjects with schizophrenia^32^ it is still open to conjecture as to whether they could be brought about by changes of Zn homeostasis that could be caused by increased expression of *SLC39A12*.

There are a number of limitations to our study. In using whole cortical homogenate we do not have the data as to whether changes in *SLC39A12* expression may be limited to a certain cell type; such data would be informative as to the potential impact of *SLC39A12* on cortical function, but was beyond the scope of the current study. Although our studies in rats have the advantage of being relatively long (12 months), they have the limitation of small treatment groups (*n*=5). Further studies in larger cohorts with a broader range of antipsychotic drugs, including atypical drugs, would be worthwhile to determine whether changes in *SLC39A12* expression may be part of the mechanism of action of such drugs.

As stated earlier, ZnT are divided into two families that contain 21 members; the ZRT-IRT-like proteins (*SLC39*; Zrt-, Irt-like Proteins) and ZnT (*SLC30*). One of our microarray studies^7^ revealed that of the 21 ZnT that were interrogated using the U133 Plus 2.0 array there were small but significant changes in levels of SLC39A1, SLC39A12, SLC30A3, and SLC30A10 mRNA in subjects with schizophrenia with effect between −4.0% and 4.6%, respectively. If, like *SLC39A12*, changes in other ZnT prove to be limited to subjects with schizophrenia and widespread throughout the cortex, then the combined effect of these changes could have substantial effects on Zn homeostasis and this could be a factor contributing to the pathophysiology of the disorder. In addition, it is not known whether the two variants of *SLC39A12* impact on different CNS functions, and therefore it is not possible to comment on the consequences of changes in the expression of one or both of these variants for subjects with schizophrenia. However, it is known that *Slc39a12* has roles in controlling cyclicAMP-response-element-binding protein phosphorylation and activity, neurite outgrowth as well as microtubule polymerization and stability.^13^ It would be predicted that changes in these processes in human cortex would have profound effects on functionality and such effects could contribute to the pathophysiology of schizophrenia.

## Materials and methods

### Human tissue

Consent was obtained from the Ethics Committee of the Victorian Institute of Forensic Medicine and the Mental Health Research and Ethics Committee of Melbourne Health before tissue was obtained from the Victorian branch of the Australian Brain Bank Network. Psychiatric diagnoses was made according to DSM-IV criteria by consensus consultant psychiatrists and a senior psychologist following a case history review using Diagnostic Instrument for Brain Studies.^33,34^ Following the case history review, duration of illness (DI) was calculated as the time from first contact with a psychiatric service. Postmortem interval and brain pH were determined as described previously.^35^

Tissue was obtained from 30 subjects with schizophrenia, made up of 15 subjects with MRDS and 15 subjects who had schizophrenia but did not have a marked loss of cortical CHRM1 (non-MRDS).^14^ Tissue was also obtained from 30 subjects with no history of psychiatric illness (controls) matched as closely as possible for age, sex, brain pH, and postmortem interval to those with schizophrenia (Table 1, full details Supplementary Table S3). In addition, levels of *SLC39A12* mRNA was measured in BA 9 from 10 subjects with MDD, 10 subjects with BD, and 9 age- and sex-matched controls (Table 1, full details Supplementary Table S3). Sample sizes were dictated by availability of tissue but experience shows they allow the detection of differences between groups ⩾15%.

All cortical regions studied were taken from the left hemisphere, with BA 9 being taken as the lateral surface of the frontal lobe, including the middle frontal gyrus superior to the inferior frontal sulcus, BA 8 being taken as the superior frontal gyrus extending from the cingulate sulcus on the medial surface to the middle frontal gyrus laterally, and BA 44 being taken as the operculum region of the inferior frontal gyrus between the ascending limb of the lateral sulcus and the inferior precentral sulcus.

### Rat tissue

The rat tissue used in our studies came from groups of five 6-week-old male Sprague-Dawley rats that were treated with vehicle, 1.0 mg/kg per day haloperidol, 10 mg/kg per day chlorpromazine, or 10 mg/kg per day thioridazine (*n*=5 per treatment arm) in drinking water for 12 months.^36^ These typical antipsychotic drugs were selected on the basis that they had been used to treat subjects with schizophrenia from whom tissue was taken to study of *SLC39A12* mRNA in human cortex (Supplementary Table S3). The doses of antipsychotic drugs were chosen to give a rat dopamine D2 receptor occupancy of between 60 and 80%, the presumed clinically effective receptor occupancy for such drugs in human.^37^ All studies were approved by the Animal Ethics Committee of the Florey Institute for Neuroscience and Mental Health. The rat cortex used in thus study was taken from the right hemisphere +2.7mm relative to bregma.

For all experiments measurements were made by individuals who were blind to diagnoses (human) and treatments (animal) and for the rat studies randomization for processing tissue were not used.

### RNA purification and first-strand cDNA synthesis

Total RNA was isolated from ~100 mg frozen tissue samples using 1.0 ml TRIzol reagent (Thermo Fisher, Scoresby, Victoria, Australia) and its quality assessed as described previously.^38^

### Real-time PCR

qPCR was performed as described previously.^38^ Primer sequences are given in Supplementary Table S4; the identities of all amplicons were confirmed by sequencing prior to qPCR. Relative quantities were calculated using the Pfaffl method.^39^ For studies using human cortex, appropriate reference genes were selected by geNorm analysis (http://medgen.ugent.be/~jvdesomp/genorm/) of the qPCR data on levels of mRNA for nine potential reference genes identified as not changing with psychiatric diagnoses from our expression microarray data. These analyses suggested that mRNA for glyceraldehyde-3-phosphate dehydrogenase (*GAPDH*), M=1.398; peptidylprolyl isomerase A (*PPIA*), M=1.386; and alpha synuclein (*SNCA*), M=1.407 had a level of stability expected of reference genes. A similar process identified succinate dehydrogenase complex, subunit A, flavoprotein (*Sdha*); mitogen-activated protein kinase kinase 5 (*Map2k5*); and mitogen-activated protein kinase 6 (*Mapk6*) as having levels of stability expected of reference genes in rat cortex. Analysis of minimally derived data (i.e., Ct corrected for qPCR efficiency) showed that levels of mRNA for the selected reference genes did not vary with diagnoses in humans or drug treatment in rats and thus were suitable for use as reference genes.

### Zinc uptake

Transformation of plasmid DNA constructs and transient transfections were essentially performed according to standard protocols. Thus, GFP-tagged *Homo sapiens* *SLC39A12* transcript variants 1 and 2, (Origene, Rockville MD, USA) were transformed into One shot Top 10 electrocomp *E.coli* cells (Thermo Fisher) by electroporation, using the Gene Pulser II electroporation system (BioRad, Gladesville, NSW, Australia), according to manufacturers’ instructions. Plasmid DNA was isolated with plasmid midi kits (Qiagen, Chadstone, Victoria, Australia), according to the manufacturer’s protocol and its concentration determined with a Nanodrop.

CHO-K1 cells were maintained in Basal Medium Eagle (BME) media supplemented with 1× penicillin/streptomycin, 2 mmol/l L-glutamine (Life Technologies), 10% foetal bovine serum (v/v) (Bovogen), 200 μmol/l L-proline and 1 mmol/l sodium pyruvate (Sigma-Aldrich, Castle Hill, NSW, Australia). Cells were seeded at 1×10^5^ cells per well 24 h prior to transfection. Plasmid DNA (1.6 μg) and Lipofectamine (4 μg) were diluted separately in 100 μl of Opti-MEM-reduced serum medium (Invitrogen), incubated for 5 min at room temperature, then mixed and incubated for 20 min at room temperature. The DNA/lipid mixture (200 μl) was added to each well, where BME had been replaced by 800 μl of fresh Opti-MEM supplemented with 10% fetal bovine serum and incubated for 24 h at 37 °C in 5% _CO2_. This protocol gave transfection efficiencies of 30% for both variants. Cells that underwent this process without plasmid DNA were used as the negative control.

^65^Zn-uptake experiments were adapted from previous methods.^40^ As the affinity of human SLC39A12 for Zn was unknown, uptake was determined at 10 and 100 μM Zn. Cells were grown in Basal Medium Eagle supplemented with 10% foetal bovine serum for 24 h. Uptake media was Hanks balanced salt solution, supplemented with 0.04 MBq of ^65^ZnCl_2_ (Perkin-Elmer, Waltham, MA, USA), made to the final concentration (10 or 100 μmol/l) with ZnCl_2_. Cells were incubated in uptake media for 10 min. The media was removed, cells washed thrice with ice-cold, Zn-free Hanks balanced salt solution (+5 mmol/l EDTA) and collected in 0.1% SDS+2 mmol/l EDTA. ^65^Zn was measured using a γ-counter (1282 CompuGamma, LKB Wallac) and standardized to total protein.

### Statistical analysis

In the case of non-experimental data and the Zn-uptake experiments, Student’s *t*-test or one-way analysis of variance (followed by Holm–Sidak’s multiple comparisons test corrected for multiple comparisons and accounting for confidence intervals and significance to identify the source of variance) to avoid an over representation of false-positive variation in data. Fisher’s Exact, or the *χ*^2^, test were used to compare non-numeric demographic data.^41^ By contrast, analyses of the qPCR data normalized to the geometric mean of the reference genes was showed that data from the different diagnostic cohorts in individual cortical regions was not normally distributed in 58% of the data sites. Hence these data were analyzed using Mann–Whitney *U*-test or the Kruskal–Wallis test with a Dunnett’s *post hoc* comparing diagnosis with controls. The qPCR data were expressed normalized to the geometric mean of levels of mRNA for three reference genes,^42^ measured within each region and hence when examining the data for variation between diagnoses, comparisons were made within each cortical region. Potential relationships with the demographic, tissue collection, and treatment data with the experimental data were assessed using a Spearman’s Rank Order Correlations. The effects of potential non-numeric confounds such as sex, genotype, suicide, and anticholinergic medication were assessed using either Mann–Whitney *U-*test or the Kruskal–Wallis test. To determine the impact of any potential confounds shown to differ significantly between diagnoses or to result in a Spearman’s *r* that deviated significantly from zero, the data from the diagnostic cohorts were compared using Rank Analyses of Variance with appropriate variables included as covariates.^43^ Analyses were performed using GraphPad Prism 6.02 (GraphPad Software, La Jolla, CA, USA) or Minitab Statistical Software release 16 (Minitab Inc., La Jolla, CA, USA).

## Figures and Tables

**Table 1:** A summary of the demographic, CNS collection, and pharmacological history data (mean±s.e.m.) for cases used in this study

*Diagnoses*	*Age (years)*	*Sex*	*DI (years)*	*Suicide*	*PMI (h)*	*pH*	*FRADD*	*LEAP*	*Anti-Ch*	*Benz*	*RIN*		
											*BA 8*	*BA 9*	*BA 44*
Controls	49±3	24M/6F	N/A	0Y/20N	44±3	**6.36**±**0.03**	N/A	N/A	N/A	N/A	6.0±0.2	8.9±0.1	7.8±0.23
Schizophrenia	48±3	25M/5F	22±2.8	8Y/22N	41±2.3	**6.26**±**0.04**	601±99	12.9±3.1	13Y/17N	12Y/18N	6.0±0.29	8.8±0.1	7.8±0.16
*P*	0.91	1.00			0.41	**0.03**					0.92	0.52	0.74
MRDS	48±4.1	13M/2F	22±4.1	4Y/11N	39±2.7	6.30±0.04	642±154	15±5.0	7Y/8N	7Y/8N	5.9±0.42	8.7±0.16	7.8±0.25
Non-MRDS	49±4.5	12M/3F	22±4.0	4Y/11N	42±4.0	6.23±0.06	553±124	11±3.8	6Y/9N	5Y/10N	6.2±0.40	8.8±0.1	7.9±0.2
d.f.	2,57				2,57	2,57					2,57	2,57	2,34
*P*	0.99	0.85	0.94	1.00	0.67	0.06	0.66	0.48	1.00	0.71	0.80	0.78	0.94
Mood disorders													
Controls	62±4.1	6M/4F	N/A	0Y/9N	47±5.4	**6.29**±**0.08**	N/A	N/A	N/A	N/A		6.2±0.5	
MDD	62±5.0	6M/4F	17.60±3.4	8Y/2N	39±5.0	**6.58**±**0.06**	305±305	1.5±1.5	10N/0Y	2Y/8N		6.7±0.27	
BD	60±4.0	6M/4F	20.30±4.1	3Y/7N	31±4.5	**6.35**±**0.06**	158±51	0.75±0.37	8N/2Y	4Y/6N		5.7±0.52	
d.f.	2,27				2,27	**2,27**						2,26	
*P*	0.88	1.00	0.62	0.07	0.08	**<0.0001**	0.5	0.62	0.47	0.62		0.30	
Cont versus MDD						**0.001**							
Cont versus BD						**NS**							
MDD versus BD						**0.001**							

Abbreviations: Anti-Ch, treated with anticholinergic drugs; BD, bipolar disorders; Benz, treated with benzodiazepines; DI, duration of illness; F, female; FRADD, final recorded antipsychotic drug dose converted to chlorpromazine equivalents; LEAP, lifetime exposure to antipsychotic drugs converted to chlorpromazine equivalents×10^−3^; M, male; MDD, Major Depressive Disorders; MRDS, Muscarinic Deficit Schizophrenia; N, No; N/A, not applicable; Non-MRDS, subjects with schizophrenia without a deficit in cortical muscarinic M1 receptors; NS, non-significant; PMI, postmortem interval; RIN, RNA integratory number; Y, yes.

Bolded entries show significant variations with diagnoses.

**Table 2:** Levels of *SLC39A12* variant 1 and 2 mRNA in Brodmann’s area 9 from subjects with MDD, BD, and age- and sex-matched controls

	*Variant 1*			*Variant 2*		
	*Controls*	*MDD*	*BD*	*Controls*	*MDD*	*BD*
Median	0.89	0.34	0.83	0.45	0.26	0.32
25% percentile	0.14	0.09	0.30	0.16	0.12	0.15
75% percentile	1.72	0.47	1.18	0.98	0.50	0.59
KW	3.1			0.7		
*P*	0.21			0.69		

Abbreviations: BD, bipolar disorders; MDD, major depressive disorders.

**Table 3:** Levels of *Slc39a12* variant 1 mRNA (ratio geometric mean of 3 reference genes) in the cortex of rats treated for 12 months with either vehicle, haloperidol (1.0 mg/kg/day), chlorpromazine (10 mg/kg/day), or thioridazine (10 mg/kg/day)

	*Vehicle*	*Haloperidol*	*Chlorpromazine*	*Thioridazine*
Median	0.53	0.59	0.54	0.59
25% percentile	0.44	0.46	0.52	0.44
75% percentile	0.57	0.67	1.03	0.62
KW	1.99			
*P*	0.57			
