# Supplementary material for: Increased cortical expression of the zinc transporter SLC39A12 suggests a breakdown in zinc cellular homeostasis as part of the pathophysiology of schizophrenia
Source: NPJ Schizophr. 2016 Mar 9;2:16002–. doi: 10.1038/npjschz.2016.2 (PMC4898896; doi:10.1038/npjschz.2016.2)
Supplement: Supplementary Table 1 [file npjschz20162-s1.doc]

Supplementary Table 1: Relationships between potential confounding factors and levels *SLC39A12* Variant 1 and 2 mRNA in the cortex of subjects with schizophrenia and age / sex matched controls.

| SLC39A12 Variant | BA |  | Age (yr) | PMI (hr) | pH | DI (yr) | FRADD | LEAP | RIN |
| --- | --- | --- | --- | --- | --- | --- | --- | --- | --- |
|  |  |  |  |  |  |  |  |  |  |
| 1 | 8 | r | 0.065 | -0.130 | **-0.334** | 0.147 | 0.133 | 0.125 | -0.015 |
|  |  | p | 0.31 | 0.16 | **0.005** | 0.22 | 0.25 | 0.26 | 0.46 |
|  |  | 95% CI From | -0.1991 | -0.3786 | **-0.5474** | -0.2358 | -0.2633 | -0.2572 | -0.2746 |
|  |  | To | 0.321 | 0.1353 | **-0.07997** | 0.4902 | 0.4911 | 0.4728 | 0.2475 |
|  |  |  |  |  |  |  |  |  |  |
|  | 9 | r | -0.093 | 0.040 | **-0.348** | -0.091 | 0.117 | -0.142 | **-0.259** |
|  |  | p | 0.24 | 0.38 | **0.003** | 0.32 | 0.28 | 0.23 | **0.02** |
|  |  | 95% CI From | -0.3459 | -0.2237 | **-0.558** | -0.446 | -0.2781 | -0.4867 | **-0.4874** |
|  |  | To | 0.172 | 0.2977 | **-0.09507** | 0.2886 | 0.4789 | 0.2402 | **0.001908** |
|  |  |  |  |  |  |  |  |  |  |
|  | 44 | r | -0.092 | **-0.240** | **-0.453** | -0.196 | 0.280 | 0.007 | 0.250 |
|  |  | p | 0.24 | **0.03** | **0.0001** | 0.15 | 0.07 | 0.48 | 0.06 |
|  |  | 95% CI From | -0.3455 | **-0.4711** | **-0.6383** | -0.5276 | -0.1159 | -0.3636 | -0.06754 |
|  |  | To | 0.1725 | **0.02304** | **-0.2172** | 0.1876 | 0.5984 | 0.3763 | 0.5216 |
|  |  |  |  |  |  |  |  |  |  |
|  |  |  |  |  |  |  |  |  |  |
| 2 | 8 | r | 0.020 | -0.221 | **-0.529** | -0.187 | -0.061 | -0.221 | -0.016 |
|  |  | p | 0.44 | 0.05 | **< 0.0001** | 0.16 | 0.38 | 0.12 | 0.45 |
|  |  | 95% CI From | -0.2426 | -0.456 | **-0.6943** | -0.5207 | -0.4337 | -0.5461 | -0.2761 |
|  |  | To | 0.2793 | 0.04228 | -0.311 | 0.1967 | 0.3299 | 0.1625 | 0.246 |
|  |  |  |  |  |  |  |  |  |  |
|  | 9 | r | 0.012 | -0.082 | **-0.440** | -0.064 | -0.344 | **-0.478** | 0.039 |
|  |  | p | 0.46 | 0.27 | **0.0002** | 0.36 | 0.03 | **0.0037** | 0.38 |
|  |  | 95% CI From | -0.2498 | -0.3359 | **-0.63** | -0.424 | -0.6427 | **-0.7208** | -0.2243 |
|  |  | To | 0.2724 | 0.183 | **-0.2040** | 0.3132 | 0.4444 | **-0.1318** | 0.2972 |
|  |  |  |  |  |  |  |  |  |  |
|  | 44 | r | -0.032 | **-0.310** | **-0.491** | -0.127 | 0.293 | -0.053 | 0.226 |
|  |  | p | 0.4 | **0.0079** | **< 0.0001** | 0.25 | 0.06 | 0.39 | 0.08 |
|  |  | 95% CI From | -0.2904 | **-0.5285** | **-0.6667** | -0.4746 | -0.1018 | -0.4151 | -0.09331 |
|  |  | To | 0.2312 | **-0.5339** | **-0.2639** | 0.255 | 0.6076 | 0.3229 | 0.5025 |

PMI = postmortem interval (hours); pH = brain pH; RINs = RNA Integrity Numbers; DI = duration of illness (years); FRADD = last recorded antipsychotic dose in chlorpromazine equivalents (mg); LEAP = lifetime exposure to antipsychotic drugs in chlorpromazine equivalents.
