# Supplementary material for: Increased cortical expression of the zinc transporter SLC39A12 suggests a breakdown in zinc cellular homeostasis as part of the pathophysiology of schizophrenia
Source: NPJ Schizophr. 2016 Mar 9;2:16002–. doi: 10.1038/npjschz.2016.2 (PMC4898896; doi:10.1038/npjschz.2016.2)
Supplement: Supplementary Table 2 [file npjschz20162-s2.doc]

Supplementary Table 2: Relationships between potential confounding factors and levels *SLC39A12* Variant 1 and 2 mRNA in the cortex of subjects with mood disorders and age / sex matched controls.

| **VARIANT 1** | |  |  |  |  |  |  |
| --- | --- | --- | --- | --- | --- | --- | --- |
|  | Age  (yr) | PMI  (hr) | pH | RIN | DI  (yr) | FRADD | LEAP |
| r | 0.212 | -0.283 | **-0.579** | -0.070 | -0.400 | 0.314 | 0.212 |
| p | 0.27 | 0.14 | **0.001** | 0.72 | 0.08 | 0.56 | 0.37 |
| 95% CI from | -0.1781 | -0.5958 | **-0.7844** | -0.4409 | -0.722 |  | -0.2679 |
| to | 0.5451 | 0.1045 | **-0.2591** | 0.3219 | 0.0668 |  | 0.607 |
|  |  |  |  |  |  |  |  |
| **VARIANT 2** | |  |  |  |  |  |  |
|  |  |  |  |  |  |  |  |
| r | 0.3671 | -0.2858 | **-0.5769** | -0.1325 | -0.2388 | -0.0857 | 0.1087 |
| p | 0.05 | 0.12 | **0.0008** | 0.49 | 0.31 | 0.92 | 0.65 |
| 95% CI from | -0.0032 | -0.593 | **-0.7803** | -0.4847 | -0.6248 |  | -0.363 |
| to | 0.6489 | 0.0941 | **-0.2631** | 0.2566 | 0.2411 |  | 0.535 |

PMI = postmortem interval (hours); pH = brain pH; RINs = RNA Integrity Numbers; DI = duration of illness (years); FRADD = last recorded antipsychotic dose in chlorpromazine equivalents (mg); LEAP = lifetime exposure to antipsychotic drugs in chlorpromazine equivalents.
