# Supplementary material for: Increased cortical expression of the zinc transporter SLC39A12 suggests a breakdown in zinc cellular homeostasis as part of the pathophysiology of schizophrenia
Source: NPJ Schizophr. 2016 Mar 9;2:16002–. doi: 10.1038/npjschz.2016.2 (PMC4898896; doi:10.1038/npjschz.2016.2)
Supplement: Supplementary Table 3 [file npjschz20162-s3.pdf]

Supplementary Table 3: Demographic and collection data for the cohort used to determine genotype and expression levels of ZIP12.

Control		Age (yrs)	Sex	PMI (hrs)	pH	Genotype: rs10764176; rs2478568; AIM	Cause of death	Anti-cholinergic	Suicide	Benzodiazepines
C1		76	M	56	6.27	A; G; CEU	Ruptured stomach ulcer	N	N	N
C2		36	M	35	6.46	A; A; CEU	Chronic obstructive airways disease	N	N	N
C3		44	M	44	6.38	GA; GA; CEU	Acute myocardial infarct	N	N	N
C4		36	M	42	6.46	A; A; CHB	Crush accident	N	N	N
C5		49	M	26	6.38	GA; G; CEU	Ruptured aneurysm	N	N	N
C6		26	M	40.5	6.41	A; GA; CEU	Drowning	N	N	N
C7		23	M	36	6.13	GA; GA; CEU	Aacute asthma	N	N	N
C8	*	71	M	50	6.33	A; A; CEU	Ischaemic heart disease	N	N	N
C9		62	M	62.5	5.71	A; A; CEU	Thoracic aorta disease	N	N	N
C10	*	59	F	20.5	6.58	GA; A; CEU	Congestive cardiac failure	N	N	N
C11		31	M	40.5	6.22	GA; A; CEU	Ischaemic heart disease	N	N	N
C12		29	F	34	6.38	GA; GA; CEU	Drog toxicity	N	N	N
C13		65	M	40	6.42	GA; GA; CEU	Ischaemic heart disease	N	N	N
C14		34	M	15.5	6.4	G; A; CEU	Ischaemic heart disease	N	N	N
C15	*	65	M	41	6.56	A; A; CEU	Ischaemic heart disease	N	N	N
C16	*	73	F	28	6.37	GA; GA; CEU	Cancer: uterus	N	N	N
C17	*	68	F	38	6.32	; A; CEU	Acute asthma	N	N	N
C18		38	F	50.5	6.14	GA; GA; CEU	Ischaemic heart disease	N	N	N
C19		29	M	43.5	6.48	A; A; CEU	Electrocution	N	N	N
C20		33	M	39	6.51	A; GA; CEU	Ruptured thoracic aorta	N	N	N
C21		29	M	15	6.46	A; GA; CEU	Congestive cardiac failure	N	N	N
C22		37	M	47	6.4	GA; GA; CEU	Cardiomyopathy	N	N	N
C23	*	50	M	69	6.43	A; GA; CEU	Ischaemic heart disease	N	N	N
C24		62	M	66	6.5	A; GA; CEU	Acute myocardial infarct	N	N	N
C25	*	65	M	20.5	6.47	GA; GA; CEU	Acute myocardial infarct	N	N	N
C26	*	50	M	65	6.4	GA; GA; CEU	Ischaemic heart disease	N	N	N
C27		35	M	27	6.4	GA; A; CEU	Acute myocardial infarct	N	N	N
C28	*	25	M	50	6.48	A; G; CEU	Exsanguination	N	N	N
C30	*	53	M	44.5	6.56	A; ; CEU	Ischaemic heart disease	N	N	N
C31		62	F	40	6.45	GA; A; CEU	Ischaemic heart disease	N	N	N
C32		22	F	42	6.31	GA; A; CEU	Multiple injuries	N	Y	N
C33	*	22	M	51	6.58	G; A; CEU	Exsanguination	N	N	N
C34	*	80	F	55	6.28	; ; CHB	Ischaemic heart disease	N	N	N
C35		27	M	30.5	6.35	; GA; CEU	Asthma	N	N	N
C36	*	36	F	60	6.4	A; A; CEU	Dilated cardiomyopathy	N	N	N
C37	*	46	M	46.5	6.08	A; GA; CEU	Acute myocardial infarct	N	N	N
C38		35	M	31	6.36	A; GA; CEU	Ischaemic heart disease	N	N	N
C39	*	22	M	62	6.39	GA; A; CEU	Iatrogenic haemorrhage	N	N	N
C40	*	25	M	35	6.15	A; GA; CEU	Ventricular hypertrophy	N	N	N
C41		63	M	38	6.37	GA; GA; CEU	Ischaemic heart disease	N	N	N
C42		69	M	38	6.37	GA; GA; CEU	Ischaemic heart disease	N	N	N
C43		60	M	27.5	6.37	A; GA; CEU	Pulmonary embolus	N	N	N
C44	*	26	M	46.5	6.37	GA; A; CEU	Electrocution	N	N	N
C45		72	M	45	5.6	GA; GA; CEU	Pyelonephritis	N	N	N
C46	*	42	M	63	6.34	A; A; CHB	Cardiomegaly	N	N	N
C47		35	M	55	6.52	A; GA; CHB	Coronary artery atheroma	N	N	N
C48	*	66	F	43	6.37	A; G; CEU	Acute myocardial infarct	N	N	N

C49		48	M	52	5.99	A; G; CEU	Ischaemic heart disease				N	N	N
C50		21	F	58	6.03	; ; CHB	Myocarditis				N	N	N
C51	*	43	M	45	6.25	A; GA; CEU	Drowning				N	N	N
C52		27	M	45	6.46	A; A; CHB	Drowning				N	N	N
C53		33	F	42	6.41	A; GA; CHB	Multiple injuries				N	N	N
C54		35	M	35	6.13	A; GA; CEU	Coronary artery atheroma				N	N	N
C55		32	F	56	6.16	GA; GA; CEU	Coronary artery atheroma				N	N	N
C56		21	M	40	5.82	A; GA; CEU	Acute epiglottitis				N	N	N
C57	*	26	M	24	6.42	A; GA; CEU	Electrocution				N	N	N
C58		30	M	27	5.86	GA; A; CEU	Coronary artery atheroma				N	N	N
C59		42	M	43	6.61	; ; CEU	Coronary artery atheroma				N	N	N
C60		38	M	44	6.19	A; A; CEU	Coronary artery atheroma				N	N	N
C61	*	48	M	24	6.37	G; A; CEU	Coronary artery atheroma				N	N	N
C62	*	72	M	39	6.21	A; GA; CEU	Coronary artery atheroma				N	N	N
C63		57	M	27	6.43	A; G; CEU	Ischaemic heart disease				N	N	N
C64		56	F	24	5.88	GA; GA; CEU	Pericardiac tamponade				N	N	N
C65	*	48	M	56	6.38	A; GA; CEU	Ischaemic heart disease				N	N	N
C66	*	68	M	41	6.06	A; G; CEU	Aortic stenosis				N	N	N
C67	*	47	F	24	5.89	A; GA; CEU	Pulmonary embolus				N	N	N
C68	*	68	M	69	6.59	A; GA; CEU	Coronary artery atheroma				N	N	N
C69	*	52	M	22	5.98	GA; A; CEU	Pulmonary embolus				N	N	N
C70		38	M	46	6.42	A; G; CEU	Trauma/asphyxia				N	N	N
C71		30	M	24	6.46	GA; A; CEU	Electrocution				N	N	N
C72		67	M	32	6.14	A; GA; CEU	Acute myocardial infarct				N	N	N
C73	*	43	M	51	6.43	G; A; CEU	Coronary artery atheroma				N	N	N
C74	*	53	M	12	6.34	GA; G; CHB	Pulmonary thromboembolism				N	N	N
C75	*	39	F	65	6.38	A; A; CEU	Mitral valve prolapse				N	N	N
C76	*	42	M	26	6.32	A; G; CEU	Coronary artery atheroma				N	N	N
C77		38	F	52	6.26	A; A; CEU	Pulmonary thromboembolism				N	N	N
C78		77	F	17	6.32	GA; GA; CEU	Hypertensive heart disease				N	N	N
C79	*	70	M	59	6.11	A; A; CEU	Ruptured ventricle				N	N	N
C80		58	M	48.5	6.41	GA; GA; CEU	Cardiomegaly				N	N	N
C81	*	75	F	53	6.01	A; A; CEU	Multiple organ failure				N	N	N
C82		65	M	36.5	6.12	A; GA; CEU	Pulmonary embolus				N	N	N
C83	*	75	M	69.4	6.19	GA; GA; CEU	Cardiogenic shock				N	N	N
C84		53	M	31.8	6.75	A; GA; CEU	Ventricular fibrillation				N	N	N
C85		42	M	26.5	6.71	GA; GA; CEU	Acute myocardial infarct				N	N	N
C86		58	M	51.5	6.16	; ; CEU	Haemorrhagic shock				N	N	N
C87	*	55	M	30.5	6.69	G; A; CEU	Coronary artery atherosclerosis				N	N	N
C88	*	52	M	33.75	6.52	; ; CEU	Cardiomegaly				N	N	N
C89		66	F	49.25	6.44	A; GA; CEU	Infrarenal atherosclerosis				N	N	N
C90		52	M	50	6.78	A; GA; CEU	Ischaemic heart disease				N	N	N
C91	*	66	M	71.75	6.47	A; A; CEU	Coronary artery atheroma				N	N	N
C92	*	42	M	30.5	6.45	GA; A; CEU	Ischaemic heart disease				N	N	N
C93		66	M	38.25	6.23	GA; A; CEU	Cardiomegaly				N	N	N
C94		57	M	48	6.65	A; GA; CEU					N	N	N
C95						A; GA; CEU					N	N	N
**Schizophrenia**		**Age (yrs)**	**Sex**	**PMI (hrs)**	**pH**	**Genotype: rs10764176; rs2478568; AIM**	**Cause of death**	**DOI (yrs)**	**Anti-psychotic**	**Chlor Eq.**	**Anti-cholinergic**	**Suicide**	**Benzodiazepines**
S1		28	M	66	6.37	; A; CEU	Drowning	10	Fluphenazine, Thioridazine, Haloperidol	1100	Y	Y	N

S2		33	M	36	6.54	; A; CEU	Hanging	14				Y	
S3		44	M	70.5	5.92	; A; CEU	Acute myocardial infarct	NR	Haloperidol, Fluphenazine	NR	N	N	N
S4		33	M	29	6.35	; GA; CEU	Overdose	8			N	Y	N
S5		30	F	43	6.48	; GA; CEU	Hanging	8	Pimozide	300	Y	Y	Y
S6	*	71	F	36	5.84	GA; GA; CEU	Chronic obstructive airways disease	48			N	N	N
S7	*	66	M	39.5	6.49	; ; CEU	Bronchopneumonia	45	Chlorpromazine, Haloperidol	1200	N	N	Y
S8		32	M	48.5	6.63	A; A; CEU	Combined drug toxicology	NR			N	Y	N
S9		20	F	75	6.34	A; GA; CEU	Asphyxia	2	Chlorpromazine	50	N	Y	N
S10		22	M	41.5	6.06	GA; A; CEU	Carbon monoxide poisoning	4	Haloperidol	1900	Y	Y	N
S11		36	M	38	6.04	GA; GA; CEU	Overdose	12	Fluphenazine	200	N	Y	Y
S12	*	51	M	20	5.98	GA; G; CEU	Ischaemic heart disease	32	Fluphenazine, Thioridazine	2000	N	N	Y
S13		57	F	42	5.98	GA; GA; CEU	Ischaemic heart disease	25	Haloperidol, Chlorpromazine	2800	Y	N	Y
S14		36	F	45	6.28	GA; GA; CEU	Carbon monoxide poisoning	4	Haloperidol	160	Y	Y	Y
S15		27	M	25	6.06	GA; GA; CEU	Alcohol poisoning	10	Haloperidol	1000	N	Y	N
S16		72	F			GA; A; CEU	Ischaemic heart disease	NR			N	N	N
S17		71	M	28.5	6.49	GA; A; CEU	Ischaemic heart disease	37	Pimozide	400	N	N	Y
S18		71	F	46.5		GA; A; CEU	Ischaemic heart disease	NR	Trifluroperazine	2000	N	N	N
S19	*	47	M	32.5	6.41	GA; A; CEU	Ischaemic heart disease	27	Fluphenazine, Thioridazine	530	N	N	N
S20	*	27	M	22	6.28	A; ; CEU	Burning	8	Chlorpromazine, Pimozide	1200	N	Y	Y
S21		30	M	53	6.25	A; G; CEU	Burning	9	Fluphenazine	300	N	Y	N
S22	*	72	F	58.5	6.48	A; G; CEU	Aspiration: Pneumonia	37	Chlorpromazine	25	N	N	N
S23		23	M	42.5	6.4	A; A; CEU	Hanging	6	Haloperidol	1750	Y	Y	N
S24		21	F	56	6.24	GA; GA; CEU	Carbon monoxide poisoning	2	Haloperidol	NR	Y	Y	N
S25	*	25	M	49	6.38	GA; A; CEU	Overdose	2	Trifluroperazine	200	N	Y	N
S26	*	53	M	37	5.98	A; A; CEU	Intestinal ischaemia	30	Fluphenazine, chlorpromazine	1700	Y	N	N
S27		63	M	45.5	5.86	A; G; CEU	Ischaemic heart disease	20			N	N	N
S28		29	M	38	6.47	A; GA; CEU	Hanging	6	Fluphenazine	1200	N	Y	N
S29		34	M	40	6.79	GA; A; CEU	Hanging	7	Trifluroperazine	200	N	Y	N
S30		59	F	46	6.44	A; G; CEU	Congestive cardiac failure	44	Fluphenazine, chlorpromazine	800	Y	N	N
S31		23	F	40.5		A; GA; CEU	Overdose	NR	Pimozide	100	N	Y	N
S32		31	M	48.5	6.56	G; A; CEU	Hanging	6	Haloperidol	830	N	Y	N
S33		40	F	61	5.71	A; G; CEU	Poisoning	24	Fluphenazine	550	Y	Y	N
S34		32	M	40	6.42	A; A; CEU	Overdose	11	Fluphenazine	150	N	Y	N
S35		22	M	48.5	6.29	GA; A; CEU	Asphyxia: Plastic bag	2	Fluphenazine, chlorpromazine	2920	N	Y	Y
S36		29	M	42	6.52	A; G; CEU	Hanging	10	Haloperidol	1000	N	Y	N
S37		38	M	36	6.44	G; A; CEU	Hanging	11	Fluphenazine	200	Y	Y	N
S38		63	M	73	6.07	GA; GA; CEU	Congestive cardiac failure	44	Trifluroperazine, chlorpromazine	300	N	N	N
S39		27	M	46	6.41	GA; GA; CEU	Hanging	8	Chlorpromazine	600	N	Y	N
S40	*	67	M	21	6.46	A; A; CEU	Pneumonia	36	Fluphenazine	75	Y	N	N
S41	*	44	M	32	6.28	G; A; CEU	Ischaemic heart disease	23	Thioridazine	600	N	N	N
S42		35	M	47	6.27	A; ; CEU	Perferated gastric ulcer	17	Fluphenzine	400	N	N	N
S43	*	47	M	41.5	6.52	A; G; CEU	Multiple injuries	21	Chlorpromazine, haloperidol	1400	N	Y	Y
S44	*	22	M	37	6.07	G; A; CEU	Pericarditis	3	Trifluroperazine, flupenthixol	450	N	N	N
S45		81	F	25	6.31	A; A; CEU	Aspiration: food	40	Trifluroperazine	100	N	N	Y
S46	*	71	M	48	6.45	A; A; CEU	Aspiration: food	53	Thioridazine	150	Y	N	Y
S47		27	F	41	5.85	G; A; CEU	Asphyxia: Plastic bag	10	Haloperidol	150	Y	Y	Y
S48		32	M	17	6.05	; ; CEU	Carbon monoxide poisoning	15	Haloperidol	670	Y	Y	Y
S49	*	53	M	43	6.23	GA; GA; CEU	Aspiration: food	7	Chlorpromazine	200	N	N	N
S50		46	M	42	5.53	GA; A; CEU	Ischaemic heart disease	12	Haloperidol	160	N	N	N
S51	*	69	M	44.5	6.38	GA; A; CEU	Ischaemic heart disease	47	Trifluroperazine	100	Y	N	N
S52		67	M	48	6.25	A; GA; CEU	Cardiomyopathy	NR	Thioridazine	400	N	N	N

S53		68	F	42	5.73	A; GA; CEU	Ischaemic heart disease	40	Trifluroperazine	400	Y	N	N
S54		42	M	34.5	6.26	GA; GA; CEU	Drowning	15	Flupenthixol, chlorpromazine	610	N	Y	Y
S55		61	M	37.5	6.46	GA; GA; CEU	Ischaemic heart disease	38	Fluphenazine	745	Y	N	N
S56		34	F	42	6.23	A; GA; CEU	Drowning	2	Risperidone	1200	N	Y	N
S57	*	22	M	37	6.17	A; A; CEU	Overdose	3	Pimozide	200	N	Y	N
S58	*	38	M	50	6.02	A; A; CEU	Meningoencephalitis	4	Clozapine	100	Y	N	N
S59	*	35	F	15	6.26	GA; GA; CEU	Coronary artery thrombosis	7	Haloperidol	300	Y	N	N
S60	*	55	M	25	6.1	GA; GA; CEU	Coronary artery atheroma	33	Thioridazine	400	Y	N	Y
S61	*	48	F	52.5	6.21	A; G; CEU	Pulmonary thromboembolism	22	Fluphenazine, chlorpromazine	700	N	N	N
S62		65	M	41	6.57	A; G; CEU	Ischaemic heart disease	35	Fluphenazine	150	N	N	N
S63	*	65	M	42	6.29	A; A; CHB	Bronchopneumonia	36	Trifluoroperazine, haloperidol	460	Y	N	N
S64		31	F	27	6.27	A; A; CEU	Cardiomegaly/ clozapine toxicity	13	Clozapine	875	N	N	Y
S65	*	65	F	50	6.35	A; G; CEU	Ruptured abdominal aneurysm	18	Fluphenazine, haloperidol	550	Y	N	Y
S66		56	M	42	6.17	A; GA; CEU	Metastatic cancer	11			N	N	N
S67		38	M	40	5.52	A; GA; CEU	Mediastinitis	15	Haloperidol	160	Y	N	N
S68	*	42	M	47	6.44	A; A; CEU/CHB	Hanging	8	Haloperidol	128	Y	Y	Y
S69	*	41	M	31	6.2	A; GA; CEU	Overdose	11	Fluphenazine, trifluroperazine	500	N	Y	Y
S70		53	M	9	6.29	A; A; CEU/CHB	Coronary artery atheroma	9	Trifluroperazine, chlorpromazine	300	N	N	Y
S71		79	M	44	6.21	A; GA; CEU	Pneumonia	56	Risperidone	800	N	N	Y
S72		57	M	24	6.06	A; GA; CEU	Coronary artery atheroma	28	Fluphenazine	150	N	N	N
S73		26	F	41	6.11	A; G; CEU	Overdose	11	Remoxipride	750	N	Y	Y
S74		55	F	64	5.52	A; GA; CEU	Diabetic ketoacidosis	33	Fluphenazine	37	N	N	N
S75		19	M	43	6.22	GA; A; CEU	Unascertained	3	Haloperidol	750	Y	Y	Y
S76		69	M	48	6.44	A; G; CEU	Carbon monoxide poisoning	6	Haloperidol	650	N	Y	Y
S77	*	23	M	78	6.19	A; A; CEU	Multiple injuries	5	Haloperidol	300	Y	Y	N
S78	*	42	M	47	6.26	A; GA; CEU	Coronary artery atheroma	22	Fluphenazine	1000	Y	N	N
S79	*	26	M	52	6.39	A; G; CEU	Carbon monoxide poisoning	2	Haloperidol	500	Y	Y	Y
S80		38	F	20	6.43	A; GA; CHB	Burning	17	Fluphenazine	485	N	Y	N
S81	*	70	M	46	5.8	GA; GA; CEU	Bronchopneumonia	20			N	N	N
S82		41	M	52	6.64	GA; A; CHB	Hanging	15	Fluphenazine	166	N	Y	N
S83	*	47	F	50	6.31	GA; A; CEU	Pneumonia	20	Risperidone	600	N	N	Y
S84		30	F	48	6.37	G; A; CEU	Hanging	10	Flupenthixol, thiothixene	600	Y	Y	N
S85	*	48	M	30	6.62	A; G; CEU	Bronchopneumonia	24	Flupenthixol, thioridazine	1250	N	N	Y
S86		35	F	39	6.38	A; GA; CEU	Incised wrist injury	9	Risperidone	150	N	Y	Y
S87		79	F	26	6.27	A; A; CEU	Hypothermia	NR	Fluphenazine	330	Y	N	N
S88		45	M	68	6.48	GA; A; CEU	Hanging	12	Trifluoroperazine	300	N	Y	N
S89		21	M	41	6.51	GA; A; CEU	Hanging	1.5			N	Y	N
S90		18	M	61	6.59	GA; A; CEU	Hanging	1.5	Olanzapine	675	N	Y	N
S91		59	F	44.5	6.19	G; A; CEU	Respiratory failure	35	Clozapine	631.75	Y	N	N
S92		58	F	72	5.85	GA; A; CEU	Pleural metastasis	8			N	N	N
S93		54	M	35.5	6.49	A; A; CEU	Hanging	38	Olanzapine, amisulpride	NR	N	Y	Y
S94		65	M	56	6.41	GA; GA; CEU	Pneumonia	46	Olanzapine	270	N	N	N
S95		58	M	42.5	6.63	; ; CEU	Ischaemic heart disease	36	Zuclopenthixol	506.25	N	N	N
S96		66	M	43.5	6.19	A; GA; CEU	Ischaemic heart disease	30			N	N	N
S97		30	M	19	6.65	A; G; CEU	Hanging	NR	Zuclopenthixol, amisulpride	1575	Y	Y	Y
S98		41	M	32	6.39	; ; CEU	Incised neck injury	18	Clozapine	NR	N	Y	N
S99		56	M	49.5	6.14	GA; A; CEU	Cardiomegaly	25	Zuclopenthixol	450	N	N	N
S100		59	M	27.6	5.89	A; GA; CEU	Ischaemic heart disease	2			N	N	N
S101		25	M	56	6.67	G; A; CEU	Hanging	NR	Risperidone	285	N	Y	N
S102		76	F	52	6.28	GA; GA; CEU	Aspiration: food	NR			N	N	N
S103		52	M	69.25	6.41	A; G; CEU	Cardiomegaly	18	Olanzapine	540	N	N	Y

S104		54	M	46.75	6.08	GA; GA; CEU	Pneumonia	NR			N	N	Y
S105		82	F	46.5	6.13	A; A; CEU	Cardiomegaly	NR			N	N	N
S106		47	M	31.75	6.62	A; GA; CEU							
S107		35	M			; ; CEU							

**Bipolar Disorder**		**Age (yrs)**	**Sex**	**PMI (hrs)**	**pH**	**Genotype: rs10764176; rs2478568; AIM**	**Cause of death**	**DOI**	**Mood Stabiliser**	**Chlor Eq.**	**other drugs**	**Suicide**	**Benzodiazepines**
BPD1		51	F	50		GA; GA; CEU	Motor Vehicle Accident	NR	Lithium				
BPD2		57	F	44.5		; ; CEU	Hanging	NR					
BPD3		29	M	43.5		A; GA; CEU	Multiple injuries	NR				Y	N
BPD4		22	M	68		GA; A; CEU	Hanging	NR	Lithium	1000	Fluphenazine	Y	N
BPD5	*	74	F	45	6.26	A; GA; CEU	Drug toxicity	35			Fluphenazine	N	Y
BPD6		58	F	41	5.68	A; G; CEU	Ischaemic heart disease	10				N	N
BPD7		66	M	28		GA; A; CEU	Hanging	NR				Y	N
BPD8		58	F	42	6.19	GA; GA; CEU	Cardiac arrhythmia	NR		600	Chlorpromazine	N	Y
BPD9		21	M	32		A; A; CEU	Overdose	NR	Lithium	660	Imipramine	Y	N
BPD10	*	59	M	34	6.46	GA; A; CEU	Ruptured aorta	24	Lithium			N	N
BPD11	*	38	M	24	6.42	GA; GA; CEU	Carbon monoxide poisoning	3	Lithium	300	Chlorpromazine	Y	N
BPD12	*	66	M	17	6.41	A; GA; CEU	Aspiration: food	12		166	Fluphenazine	N	Y
BPD13		55	F	52	6.46	A; GA; CEU	Unacertained	14	Lithium	300	Chlorpromazine	N	Y
BPD14		60	F	50	6.08	; ; CEU	Cardiomegaly	23	Lithium	NR	Trifluroperazine	N	Y
BPD15	*	61	M	58	6.44	GA; A; CEU	Acute myocardial infarct	40	Valproate	1500	Flupenthixol	N	Y
BPD16	*	42	F	25	6.54		Hanging	20	Lithium		Venlafaxine	Y	Y
BPD17	*	79	M	8.25	6.09		Cholecystitis	17	Valproate	67.5	Olanzapine	N	N
BPD18	*	59	M	37.5	5.97		Ischaemic heart disease	8	Lithium			N	N
BPD19	*	64	F	26	6.46	A; GA; CEU	Ischaemic heart disease	8	Valproate		Venlafaxine	N	N
BPD20	*	56	F	36	6.43		Carbon monoxide poisoning	36	Carbamazepine		Venlafaxine	Y	N

**Major Depressive Disorder**		**Age (yrs)**	**Sex**	**PMI (hrs)**	**pH**	**Genotype: rs10764176; rs2478568; AIM**	**Cause of death**	**DOI (yrs)**	**Anti-depressant**	**Chlor eq**	**other drugs**	**Suicide**	**Benzodiazepines**
MDD1		39	M	44.5		GA; GA; CEU	Copper sulphate poisoning	NR			Frusemide	Y	N
MDD2		36	F	35		G; A; CEU	Overdose	NR				Y	N
MDD3		36	F	35.5	5.91	A; GA; CEU	Pulmonary embolus	NR	Amitryptaline		Lithium	N	N
MDD4		19	M	58	6.89	A; GA; CEU	Combined drug toxicology	2				Y	N
MDD5		61	F	36	6.42	GA; A; CEU	Overdose	20				Y	N
MDD6		34	F	30		A; GA; CEU	Choking	NR				?	N
MDD7		51	M	50	6.37	GA; GA; CEU	Acute alcohol toxicity	NR	Diothiepin	50	Thioridazine	N	N
MDD8		52	F	63	6.46	A; GA; CEU	Hanging	NR				Y	N
MDD9		58	F	49	6.44	A; G; CEU	Pulmonary embolus	17		270	Olanzapine	N	N
MDD10		74	M	46	6.64	A; G; CEU	Hanging	15	Sertaline			Y	N
MDD11		63	F	10.5	6.54	A; A; CEU	Overdose	NR		268	Quetiapine	Y	Y
MDD12		41	M	28	6.17	A; GA; CEU	Carbon monoxide poisoning	5					
MDD13		79	F	27	6.38	A; G; CEU	Pulmonary embolus	23	Amitryptaline			N	Y
MDD14		54	F	67.5	6.52	GA; G; CEU	Overdose	NR	Tranylcypromine	NR	chlorpromazine	Y	Y
MDD15		42	F	72	5.64	GA; A; CEU	Hepatorenal failure					Y	N
MDD16		34	M	31.5	6.48	GA; A; CEU	Hanging	NR	paroxetine			Y	Y
MDD17		54	F	67	6.51	G; ; CEU	Hanging	20				Y	N
MDD18		27	F	45	6.55	GA; A; CEU	Hanging	0.5	Fluoxetine		Lithium	Y	Y
MDD19		48	F	28.5	5.9	A; G; CEU	Overdose	NR	Fluvoxamine		alprazolam	Y	Y
MDD20		20	F	53.5	6.86	GA; A; CEU	Hanging	NR				Y	N
MDD21	*	37	M	57.75	6.84	A; G; CEU	Hanging	14	Sertaline			Y	N

MDD22	*	51	M	41	6.71	GA; GA; CEU	Hanging	15	Clomipramine, venlafaxine		Y	N
MDD23	*	50	F	50.5	6.85	; ; CEU	Combined drug toxicology	40			?	N
MDD24	*	77	F	16.7	6.49	GA; A; CEU	Overdose	26	Doxepin		Y	N
MDD25	*	69	M	44.5	6.45	A; GA; CEU	Drowning	20			Y	N
MDD26	*	79	M	24	6.32	G; A; CEU	Asphyxia	17	Nortripyline		Y	Y
MDD27	*	55	M	47.75	6.6	GA; G; CEU	Hanging	3	Venlafaxine	zolpidem	Y	N
MDD28	*	68	M	60.75	6.65	GA; GA; CEU	Hanging	9	Citalopram, mianserin		Y	Y
MDD29		62	M	37.5	6.16	A; GA; CEU	Coronary artery atheroma	NR	Venlafaxine		N	Y
MDD30	*	87	F	24.5	6.44	A; A; CEU	Chest infection	7	Sertaline	zolpiclone	N	N
MDD31	*	51	F	23.5	6.49	A; A; CEU	Drug toxicity	25	Mirtazapine	risperidone	Y	N
MDD32		87	M	56.5	6.52	GA; GA; CEU	Hanging	NR	Venlafaxine		Y	N
MDD33		64	N	36.75	6.59	GA; A; CEU	Hanging	NR	Venlafaxine		Y	N
MDD34		35	F	39	6.74	; ; CEU					Y	

PMI = post-mortem interval; DOI = duration of illness; Chlor Eq. = chlorpromazine equivalents (mg). * These subjects had SLC39A12 mRNA levels assessed.
