# Supplementary material for: Increased cortical expression of the zinc transporter SLC39A12 suggests a breakdown in zinc cellular homeostasis as part of the pathophysiology of schizophrenia
Source: NPJ Schizophr. 2016 Mar 9;2:16002–. doi: 10.1038/npjschz.2016.2 (PMC4898896; doi:10.1038/npjschz.2016.2)
Supplement: Supplementary Table 4 [file npjschz20162-s4.doc]

Supplementary Table 4: The nucleotide sequences of primers used to amplify cDNA prepared from human and rat cortex.

| **Real-time PCR** | | |
| --- | --- | --- |
| Gene target | Gene ID | Primer sequence |
| *Human* | | |
| SNCA | 6622 | Forward: ctgctgctgagaaaccaaa  Reverse: ctgctccctccactgtctt |
|  | 2597 | Forward: tgcaccaccaactgcttagc |
| GAPDH |  | Reverse: ggcatggactgtggtcatgag |
| PPIA | 5478 | Forward: atggtcaaccccaccgtgttcttcg  Reverse: cgtgtgaagtcaccaccctgacaca |
| ZIP12 variant 1 | 221074 | Forward: CACCATCTTGCACTCAACTC |
|  |  | Reverse: TTCTGTTGGAGGCTGTCATA |
| ZIP12 variant 2 | 221074 | Forward: ccaaatgacaagaaaagccc |
|  |  | Reverse: TTCTGTTGGAGGCTGTCATA |
| *Rat* | | |
| Sdha | 157074 | Forward: GTCCATACACCGAATAAGAG  Reverse: GCCAGCACCATAGATACC |
| Map2k5 | 29568 | Forward: GTAAGAAGTGACGAAGAGATGAAG  Reverse: GCTGGTGTGCTGAGATGG |
| MapK6 | 58840 | Forward: GAGAGGCAAGTCCAAGTG  Reverse: AAGGCATCAAAGTCAAAGC |
| Zip12 | 291328 | Forward: GGTGGAGGCAAGTCTATATC  Reverse: ATGAAGAAGAGAAGGCTGTG |
